# Akt Inhibition as Preconditioning Treatment to Protect Kidney Cells against Anoxia

**DOI:** 10.3390/ijms23010152

**Published:** 2021-12-23

**Authors:** Nicolas Melis, Romain Carcy, Isabelle Rubera, Marc Cougnon, Christophe Duranton, Michel Tauc, Didier F. Pisani

**Affiliations:** 1 Laboratory of Cellular and Molecular Biology, Center for Cancer Research, National Cancer Institute, Bethesda, MD 20892, USA; nicolas.melis@nih.gov; 2 Université Côte d’Azur, CNRS, LP2M, 06103 Nice, France; romain.carcy@yahoo.com (R.C.); isabelle.rubera@univ-cotedazur.fr (I.R.); cougnon@unice.fr (M.C.); duranton@unice.fr (C.D.); tauc@unice.fr (M.T.); 3 CHU Nice, Hôpital Pasteur 2, Service de Réanimation Polyvalente et Service de Réanimation des Urgences Vitales, 06103 Nice, France; 4 Laboratories of Excellence Ion Channel Science and Therapeutics, 06103 Nice, France

**Keywords:** ischemia, ROS, mitochondria, triciribine, GC7, eIF5A

## Abstract

Lesions issued from the ischemia/reperfusion (I/R) stress are a major challenge in human pathophysiology. Of human organs, the kidney is highly sensitive to I/R because of its high oxygen demand and poor regenerative capacity. Previous studies have shown that targeting the hypusination pathway of eIF5A through GC7 greatly improves ischemic tolerance and can be applied successfully to kidney transplants. The protection process correlates with a metabolic shift from oxidative phosphorylation to glycolysis. Because the protein kinase B Akt is involved in ischemic protective mechanisms and glucose metabolism, we looked for a link between the effects of GC7 and Akt in proximal kidney cells exposed to anoxia or the mitotoxic myxothiazol. We found that GC7 treatment resulted in impaired Akt phosphorylation at the Ser473 and Thr308 sites, so the effects of direct Akt inhibition as a preconditioning protocol on ischemic tolerance were investigated. We evidenced that Akt inhibitors provide huge protection for kidney cells against ischemia and myxothiazol. The pro-survival effect of Akt inhibitors, which is reversible, implied a decrease in mitochondrial ROS production but was not related to metabolic changes or an antioxidant defense increase. Therefore, the inhibition of Akt can be considered as a preconditioning treatment against ischemia.

## 1. Introduction

Ischemia/reperfusion (I/R) is characterized by an interruption in the blood supply to tissues or organs, which leads to oxygen and nutrient deprivation and then to a sudden and acute reoxygenation and nutrient availability [1,2]. This ischemic stress, which occurs in pathological situations (stroke, myocardial infarction) and during operations with voluntary blood arrest (organ transplantation, cardiovascular surgery), can lead to cell death and irreversible ischemic lesions [1]. The kidney is an organ heavily dependent on oxygen supply and is therefore extremely sensitive to I/R [3]. The first step in a kidney transplant is a temporary arrest of perfusion and blood flow, known as warm ischemia. The consecutive lack of oxygen during this step induces an anaerobic metabolism and disrupts the aerobic synthesis of ATP, which also creates oxidative stress [4]. Damage also occurs at the time of reperfusion, when an excess of oxygen generates oxidative stress again [5]. Thus, I/R generates a two-stage oxidative stress characterized by an overproduction of reactive oxygen species (ROS), which causes irreversible oxidation of biological molecules ultimately leading to cell death [6].

Oxidative stress is due to an imbalance between ROS production and detoxification. ROS are produced from various sources in the cell; most of them are produced by the mitochondria, especially during I/R [7]. In fact, depletion of oxygen and nutrients during ischemia affects the mitochondrial activity, resulting in a change in electron transport through the mitochondrial respiratory chain, leading to the production of ROS (mainly at the level of complexes I and III of the respiratory chain) and in a decrease in the ATP level [8,9,10]. During reperfusion, the sudden reactivation of mitochondrial aerobic metabolism causes an excessive function of mitochondrial complexes, which induces an increase in ROS production [11].

Combating these ischemic injuries, which are primarily due to oxidative stress, remains a major public health challenge and is made more difficult by the fact that their consequences are often a source of continuous deterioration for the well-being of patients. In recent years, the objective has been to find clinical and pharmacological treatments to reduce or prevent the occurrence of oxidative stress in ischemic pathologies. Given that transplantation is accompanied by an inevitable I/R sequence that can be expected and thus can be contained by preconditioning treatments, several pharmacological molecules have been proposed to curb the development of this stress by targeting oxidative stress and its effects.

I/R injury has been shown to be alleviated by various molecules that act on numerous pathways known to be involved in oxygen and nutrients sensing, such as the hypoxia-inducible transcription factors (HIF) pathway, nuclear factor erythroid2-related factor2 and Kelch-like ECH-associated protein 1 (NRF2/KEAP1) pathway and 5′ AMP-activated protein kinase (AMPK) pathway [12,13]. Some of these drugs were dependent on the activation of the phosphatidylinositol 3 kinase (PI3K)/protein kinase B (Akt) pathway [14]. Akt is a protein kinase ubiquitously expressed by organisms under two isoforms Akt1 and Akt2, and is known to control various cellular processes such as glucose metabolism, proliferation, and apoptosis [15]. Thus, Akt belongs to the family of pro-survival kinases named the Reperfusion Injury Salvage Kinase (RISK) pathway, which is associated with the survival and inactivation of apoptosis-associated proteins and are involved in various ischemic protective mechanisms [16]. Akt activation promotes phosphorylation of downstream molecules, including the apoptosis-related family members of Bcl2, the mammalian target of rapamycin (mTOR), glycogen synthase kinase-3 (GSK3) and various transcription factors [15,16]. Therefore, numerous publications reported that Akt activation mediates the beneficial effects of the tested compounds as well as in brain, heart, kidney and liver ischemia [17,18,19,20,21]. Several ischemic preconditioning protocols, which consist of applying brief repetition of artery occlusion and reperfusion steps, have been described as efficient for ischemic organ protection especially during transplantation [22]. Remote ischemic preconditioning involves multiple cycles of ischemia and reperfusion of limb extremities and allows protection of distant organs, such as heart and kidney [23], mainly by the production of circulating effectors [24]. In a different way, local ischemic preconditioning, by briefly repeated clamping of the organ artery [25], directly stimulates the organ to activate signaling pathways [26]. Interestingly, Akt activation seemed essential to the protective effect of both remote [27] and local ischemic preconditioning [28,29,30]. Nevertheless, some works reported that Akt had to be inactivated in order to mediate the protective effect of the agents against ischemia [31,32,33]. These conflicting results could not be surprising given the multitude of signaling pathways involved in I/R responses and oxygen sensing to respond to intracellular stress such as ROS, nitric oxide derivatives and ATP depletion. Moreover, the Akt response must depend on the metabolic phenotype of the cells as it is highly involved in glucose metabolism. Finally, it is interesting to note that, without treatment, the first tissue response to ischemia may be a decrease in Akt phosphorylation as described in heart [34,35,36] and kidney [37,38], suggesting that Akt deactivation for some cells be a first line of defense against oxygen and nutrient deprivation.

We have recently shown that inhibition of eIF5A activation/hypusination, by a pretreatment with GC7 (N1-guanyl-1,7-diaminoheptane), is able to improve ischemic tolerance at the cell and organ level [39,40,41]. eIF5A protein is activated by a post-translational modification, called hypusination, unique and extremely conserved across evolution from yeast to mammals [42]. This modification occurs on a specific lysine residue and is carried out from the polyamine spermidine by the successive action of deoxyhypusine synthase (DHPS) and deoxyhypusine hydroxylase (DOHH). eIF5A and its hypusinated form have been involved in initiation and elongation of proteins as well as in mRNA nuclear export [43]. We have previously demonstrated that GC7 (N1-guanyl-1,7-diaminoheptane), a spermidine analog specifically inhibiting DHPS activity and thus hypusination of eIF5A, allowed ischemic protection of kidney cells [40,41] and neurons [39] from mouse to human origins and even enhanced survival of fly under hypoxia [44]. Conversely, diet supplementation in polyamine, which leads to an increase in eIF5A hypusination, reduced survival of fly under hypoxia [44]. From a metabolic point of view, GC7 decreases oxidative phosphorylation, acutely and reversibly, reprograms the function and metabolism of kidney cells in order to make glucose their only substrate and, thus, enables the cells to be independent of oxygen through anaerobic glycolysis [41,45]. In addition, GC7 has been shown to decrease the oxidative stress associated with the above stresses [39,40,41]. In search of pathways involved in the GC7 anti-ischemic effect, we have found that GC7 pretreatment of kidney cells resulted in a surprising decrease in Akt phosphorylation. Anticipating the inhibition of Akt phosphorylation as a preconditioning event, we decided to test this hypothesis, and we show that Akt inhibitors effectively protect cells from anoxia as well as from myxothiazol treatment, a drug disrupting mitochondrial activity. This protection, which is reversible, appears to be related to a reduction in mitochondrial ROS production without affecting the metabolism of kidney cells. In fact, we demonstrate that even if Akt mediates several positive effects against I/R, its inhibition could be considered as a preconditioning treatment to protect cells from ischemia.

## 2. Results

### 2.1. Anoxia Rapidly Inhibits Akt Phosphorylation

**Figure 1:**
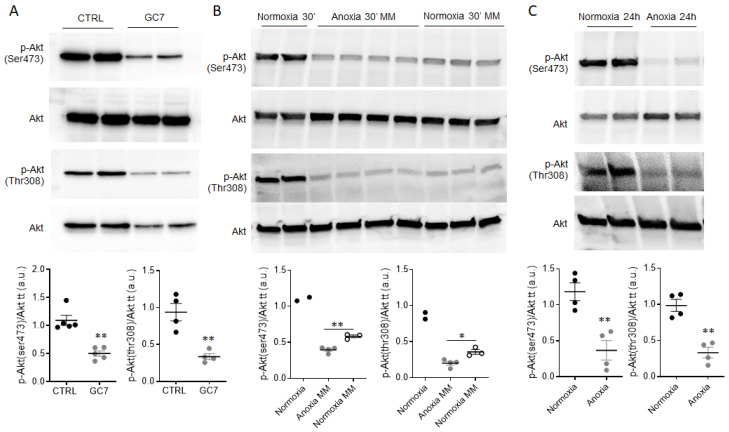
Akt phosphorylation is inhibited by anoxia and by ischemic protective treatment in kidney cells. PCT cells were (**A**) treated for 24 h with 30 µM GC7, (**B**) deprived of nutrients in minimum media (MM) under normoxic or anoxic conditions for 30 min or (**C**) submitted to anoxia for 24 h. At the end of treatment, phosphorylation of Akt on both Ser(473) and Thr(308) sites were assessed by western blot as well as total Akt. The scatter plot of the above Western blot bands quantification represents individual p-Akt/total-Akt ratios along with mean ± SEM. n = 2 to 5. * *p* < 0.05; ** *p* < 0.01.

We have previously shown the high effectiveness of GC7 pretreatment, a specific and reversible eIF5A hypusination inhibitor, in protecting the kidney from ischemia situations and proximal convoluted tubule cells (PCT) from anoxia [41]. Surprisingly, GC7 pretreatment for 24 h induced inhibition of Akt phosphorylation at both Ser473 and Thr308 sites (Figure 1A). To determine if this inhibition could be considered as a preconditioning event mimicking a stress response, PCT cells were deprived of nutrients in minimum media (MM) for 30 min either maintained under normoxia or exposed to anoxia to mimic an acute ischemic event. As shown in Figure 1B, serum deprivation induced a decrease in Akt phosphorylation on both Ser473 and Thr308 sites compared to cells receiving nutrients and oxygen for the same time. Interestingly, when cells were deprived of nutrients and exposed to anoxia, inhibition was more important. Prolonged exposure of PCT cells to anoxia for 24 h resulted in the same inhibition of Akt at the Ser473 and Thr308 phosphorylation site (Figure 1C). These results demonstrated that anoxia and nutrient deprivation rapidly inhibit Akt phosphorylation, which can reflect a possible adaptation of PCT cells to resist these stresses.

### 2.2. Inhibition of Akt Leads to Anoxia Resistance

**Figure 2:**
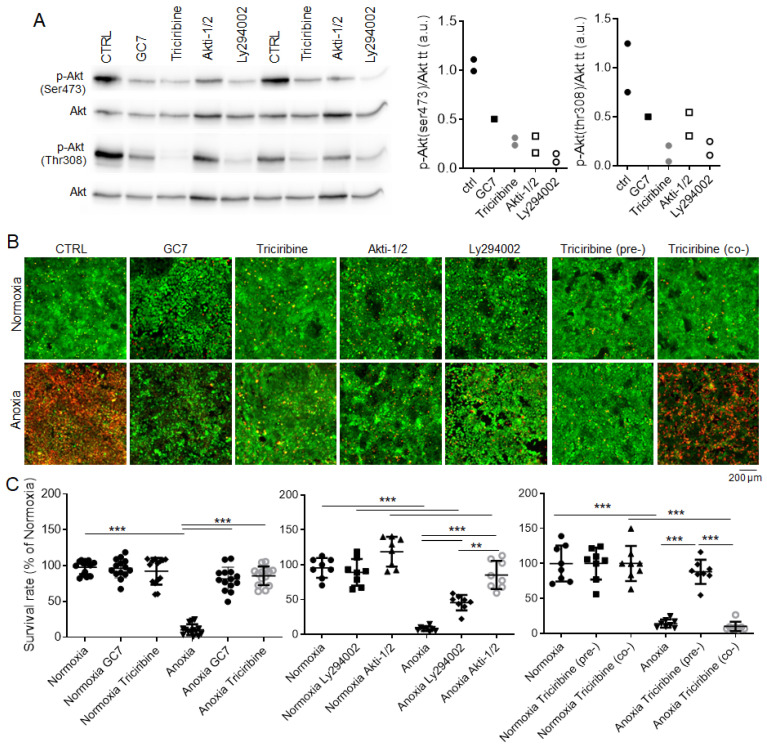
Inhibition of Akt promotes PCT cell survival under anoxia. (**A**) PCT cells were treated or not for 24 h with GC7 (30 µM) and for 30 min with triciribine (1 µM), Akti-1/2 (10 µM) and Ly294002 (50 µM). At the end of treatment, phosphorylation of Akt on both Ser(473) and Thr(308) sites were assessed by Western blot as well as total Akt. Scatter plot representation of individual p-Akt/Akt band intensities values from adjacent Western blots. (**B**,**C**) PCT cells were treated or not with GC7 (30 µM), triciribine (1 µM), Akti-1/2 (10 µM) and Ly294002 (50 µM) for 24 h. Then, cells were retreated and either maintained under normoxia or submitted to anoxia for another 24 h. triciribine (pre-): cells were treated the first 24 h before anoxia; triciribine (co-): cells were treated just at the moment of anoxia. Cell viability was then evaluated using a live/dead fluorescence assay. (**B**) Representative images of each condition with live (green, calcein-FITC) and dead (red, ethidium bromide homodimer) cells and (**C**) corresponding survival rate (live/dead cells). Scatter plot representation of individual values along with mean ± SD. n = 14 or 8. ** *p* < 0.01, *** *p* < 0.001.

To recreate Akt inhibition, we used three different compounds known to inhibit Akt phosphorylation: Akti-1/2 and triciribine are two selective and reversible inhibitors of Akt1/2 and Akt1/2/3, respectively, LY294002 is an inhibitor of the well-known Akt activator phosphatidylinositol-3-kinase (PI3K). We first evaluated the efficiency of these compounds to acutely inhibit Akt phosphorylation in our model. Thirty minutes of treatment with triciribine (1 µM), Akti-1/2 (10 µM) and Ly294002 (50 µM) displayed a strong inhibition of Akt phosphorylation on both Ser473 and Thr308 sites (Figure 2A), reproducing the effect observed after GC7 treatment for 24 h (30 µM). Of note, acute GC7 treatment (30 min) did not affect Akt phosphorylation (data not shown), strongly suggesting an indirect impact on Akt.

Using the same concentrations, confluent PCT cells were pretreated with Akt inhibitors for 24 h and then exposed to anoxia for 24 h in order to determine their survival. Untreated PCT cells displayed around 90% mortality after 24 h under anoxia, while GC7 treated cells displayed less than 10% mortality (Figure 2B,C). Surprisingly, the pre-inhibition of Akt enabled PCT cells to withstand the lack of oxygen (Figure 2B,C): cells treated with Ly294002 displayed 50% survival while Akti-1/2 treated cells survived up to 70%. riciribine was the most potent compound with only 10% mortality after anoxia (Figure 2B,C). At the used concentration, none of these compounds displayed any toxicity as all cells were alive under normoxia regardless of treatment (Figure 2B,C). To better characterize triciribine, the most potent compound, we treated PCT cells with 1 µM triciribine either 24 h before or just prior to the exposure to 24 h anoxia (Figure 2B,C). While PCT cells pretreated with triciribine were protected against anoxia, cells treated just prior anoxia (co-) died similarly to the control (Figure 2B,C). Thus, preconditioning cells by inhibiting Akt is sufficient to induce long-term resistance to anoxia similarly to GC7 pretreatment.

### 2.3. Modulation of Glucose Metabolism Does Not Participate in Anoxia Resistance

**Figure 3:**
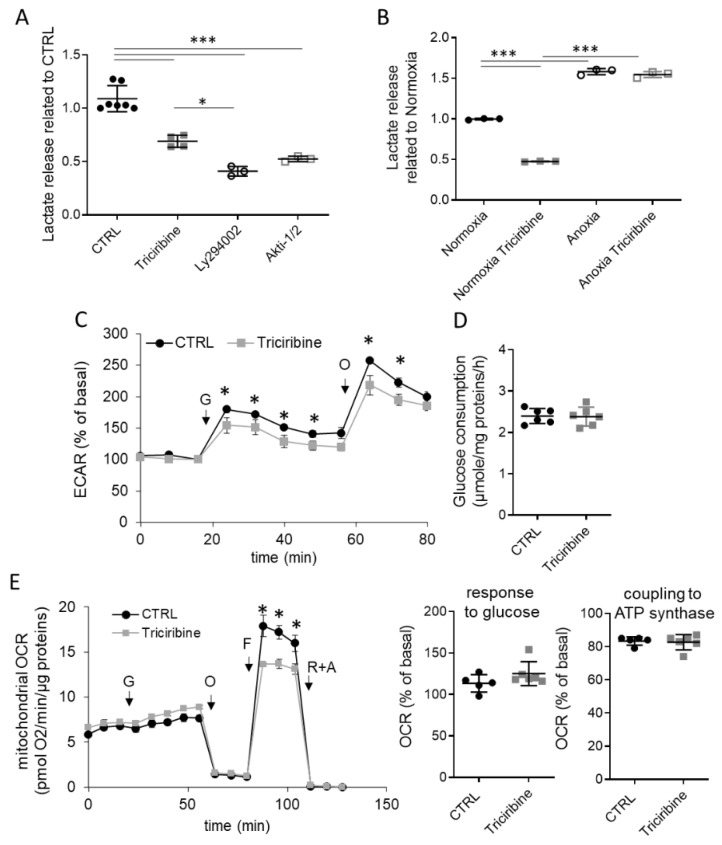
Akt inhibition affects glucose metabolism without modification of oxidative phosphorylation. PCT cells were treated or not 24 h with triciribine (1 µM), Akti-1/2 (10 µM) and Ly294002 (50 µM) and used for different analyses. (**A**,**B**) Measurement of lactate media content related to whole cell proteins content at the end of treatment (**A**) or after an additional 24-h exposition to normoxia or anoxia (**B**). (**C**) Cells were analyzed using Seahorse technology to evaluate extracellular acidification. Arrows indicate injection of the different compounds (G, glucose 10 mM; O, oligomycin A 1 µM). (**D**) Evaluation of cell glucose consumption corresponding to media glucose content decrease. (**E**) Cells were analyzed using Seahorse technology to evaluate mitochondrial oxygen consumption. Arrows indicate injection of the different compounds (G, glucose 10 mM; O, oligomycin A 1 µM; F, FCCP 1.2 µM; R + A, Rotenone + Antimycin A 2 + 2 µM). Scatter plot representation of individual values along with mean ± Sd. N = 3–7 (**A**), 3 (**B**), 5–6 (**C**–**E**). * *p* < 0.05; *** *p* < 0.001.

Adaptation to anoxia is associated with a metabolic change to promote glycolysis in the absence of oxygen, characterized by an increase in lactate release and a decrease in cellular metabolic demand, as observed with GC7 treatment [41,45]. Unexpectedly, PCT cells treated for 24 h with Akt inhibitors displayed a 50% decrease in lactate release (Figure 3A). Interestingly, this metabolic change was not maintained under anoxia. Indeed, when cells were first treated 24 h with or without triciribine and then submitted to anoxia, there was an increase in lactate release as expected regardless of treatment (Figure 3B). This demonstrated that even if the inhibition of Akt resulted in a decrease in glycolysis, it did not disrupt the normal adaptation of cells to anoxia, which corresponds to an increase in ATP synthesis through glycolysis. The decrease in lactate release was confirmed by extracellular acidification rate (ECAR) analysis, which showed less acidification in response to glucose in triciribine-treated cells compared to untreated cells (Figure 3C). Moreover, the ability of cells to perform glycolysis seemed reduced as maximal ECAR (measured after oligomycin A addition) was lower in triciribine treated PCT cells compared to control cells (Figure 3C). This difference in potential found after 24 h of treatment was not related to a decrease in glucose consumption (Figure 3D).

We then analyzed the mitochondrial activity. PCT cells were treated with triciribine for 24 h and then analyzed mitochondrial oxygen consumption rate (OCR). As expected, the addition of 10 mM glucose did not modify much mitochondrial OCR, and there was a slight but not significant increase found after triciribine treatment (Figure 3E). Indeed, PCT cells displayed mainly a glutamine-dependent oxidative metabolism, directly related to their glucose reabsorption genuine function [39]. In addition, triciribine changed neither the basal oxygen consumption in the absence of glucose nor the coupling to the ATPsynthase (measured after the addition of oligomycin A) (Figure 3E), showing that glutaminolysis and oxidative phosphorylation were unaffected by Akt inhibition. Nevertheless, triciribine treatment decreased the maximal mitochondrial respiration of PCT cells induced by FCCP addition (Figure 3E), suggesting that triciribine treatment induces a partial inhibition of mitochondrial function, normally unused under basal conditions.

### 2.4. Akt Inhibition Protects from Mitochondria-Originated ROS

**Figure 4:**
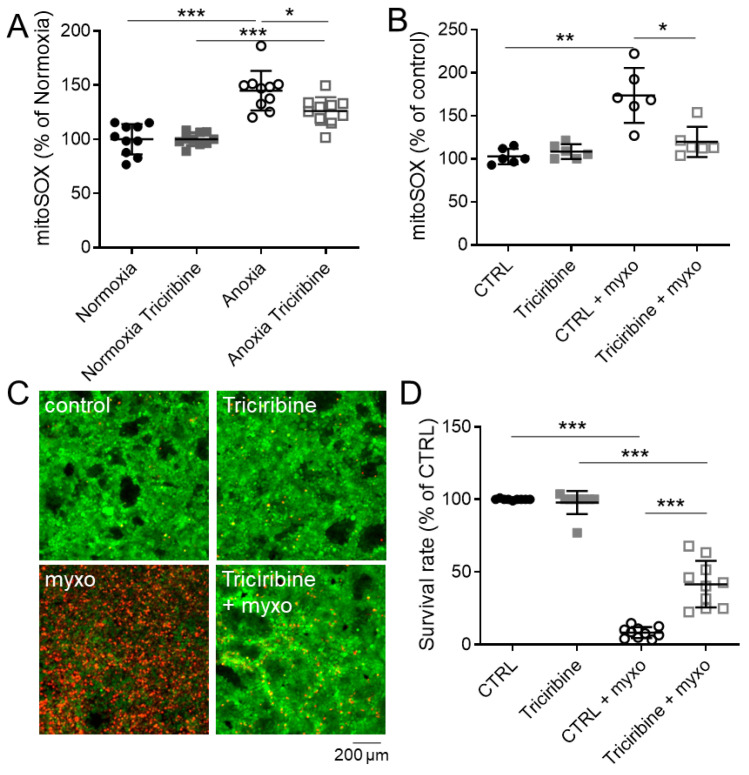
Effect of Akt inhibition on mitochondrial ROS production. (**A**,**B**) Mitochondrial ROS content evaluated with MitoSOX probe on PCT cells treated or not 24 h with triciribine (1 µM) and then submitted to anoxia for 8 h (**A**) or treated with 1 µM myxothiazol for 6 h (**B**). Fluorescence was measured on cell lysate and normalized by protein content. (**C**,**D**) Cell viability was evaluated using a live/dead fluorescence assay on PCT cells treated 24 h with triciribine (1 µM) and then with 1 µM myxothiazol for 24 h. (**C**) Representative images for each condition with live (green, calcein-FITC) and dead (red, ethidium bromide homodimer) cells and (**D**) corresponding survival rate (live/dead cells). Scatter plot representation of individual values along with mean ± Sd. n = 10 (**A**,**D**) or 6 (**B**). * *p* < 0.05; ** *p* < 0.01, *** *p* < 0.001.

Along ischemia/anoxia, decrease in oxygen and nutrient supply leads to local production of superoxide anion, a type of reactive oxygen species (ROS), within mitochondria [12]. To assess the ability of Akt inhibition to protect cells from mitochondrial ROS production, PCT cells were pretreated with triciribine for 24 h and then submitted to anoxia for 8 h. As expected, anoxia induced a significant increase in mitochondrial ROS assessed by MitoSOX fluorescence increase, a specific probe for mitochondrial ROS production (Figure 4A). Interestingly, PCT cells pretreated with triciribine displayed no difference with the control condition under normoxia and a slight but not significant increase in mitochondrial ROS production under anoxia (Figure 4A). To assess the impact of triciribine on oxidative stress generation through production of mitochondrial ROS, we exposed PCT cells to myxothiazol, an irreversible respiratory chain Complex III inhibitor. This “mitotoxic” compound disrupts mitochondrial activity, promotes mitochondrial ROS production and ultimately cell death, as found after 24 h treatment in PCT control cells (Figure 4B–D). Remarkably, triciribine pretreatment prevents superoxide anion generation following 6 h of myxothiazol treatment observed in control cells (Figure 4B) and increases cell viability by approximatively 50% following 24 h of myxothiazol treatment (Figure 4C,D). Thus, Akt inhibition protects PCT cells from myxothiazol-induced cell death by preventing mitochondrial anion superoxide generation.

### 2.5. Akt Inhibition Does Not Protect from H_2_O_2_ Overproduction

**Figure 5:**
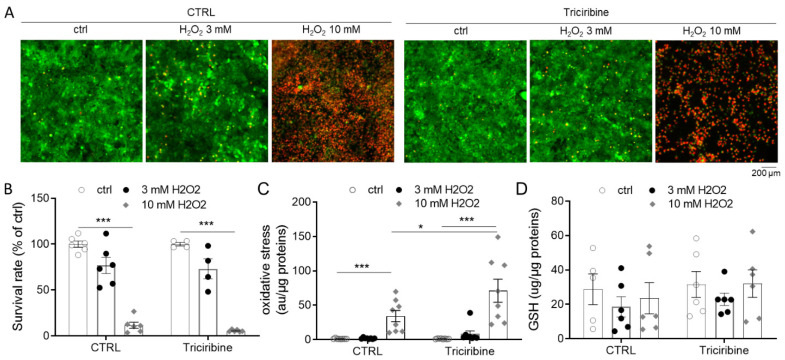
Effect of Akt inhibition on oxidative stress induced by hydrogen peroxide treatment. PCT cells treated or not for 24 h with triciribine (1 µM) and then treated with 3 or 10 mM H_2_O_2_ for 24 h. (**A**,**B**) Cell viability was evaluated using a live/dead fluorescence assay on PCT cells treated. (**A**) Representative images for each condition with live (green, calcein-FITC) and dead (red, ethidium bromide homodimer) cells and (**B**) corresponding survival rate (live/dead cells). (**C**) Oxidative stress level was assessed by the addition of CMH2DCFDA probes. Fluorescence values were evaluated on cell lysate and normalized by protein content. (**D**) GSH content normalized by protein content. Scatter plot representation of individual values along with mean ± Sd. n = 4–6 (**B**), 8 (**C**) or 6 (**D**). * *p* < 0.05; *** *p* < 0.001.

To determine whether triciribine could protect the cells from general oxidative stress in addition to mitochondrial ROS production, we exposed PCT cells to different concentrations of hydrogen peroxide (H_2_O_2_). While PCT cells were not drastically affected by 3 mM H_2_O_2_, 10 mM H_2_O_2_ treatment led to massive cell death (Figure 5A,B), linked to an elevated oxidative stress (Figure 5B), evidenced by a strong activation of the CMH2DCFDA probe, known to be sensitive to peroxynitrite (ONOO^−^) and hydroxyl radical (HO^−^), two highly reactive oxygen species. Unfortunately, triciribine pretreatment did not prevent cell death and oxidative stress generation due to H_2_O_2_ (Figure 5A–C). Likewise, triciribine did not modify the cellular content of GSH, a major antioxidant molecule, in PCT cells (Figure 5D).

### 2.6. Reversibility of Akt Inhibition Effect on PCT Cells

**Figure 6:**
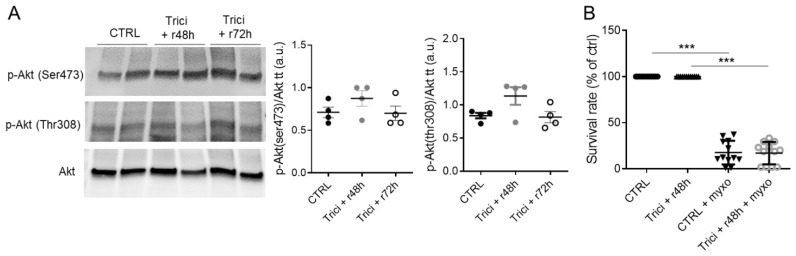
Reversibility of triciribine treatment. Akt phosphorylation and myxothiazol induced cell death were assessed after 24 h triciribine treatment (1 µM) followed by 2 washes and either a 48 (r48 h) or 72 (r72 h) h recovery period. Control cells were stopped at 48 h after media change. (**A**) Phosphorylation of Akt on both Ser(473) and Thr(308) sites were assessed by western blot as well as total Akt proteins. Representative WB and related ratio analysis of p-Akt/Akt band intensities values along with mean ± SEM. n = 3. (**B**) Control or 48 h recovery cells were treated for 24 h with 1 µM myxothiazol and survival rate was evaluated using a live/dead fluorescence assay. Scatter plot represents individual values along with mean ± SD. n = 12. *** *p* < 0.001.

We showed that triciribine pretreatment prevented anoxia or myxothiazol stress induced cell death 24 h later. To determine if the effect was sustained over time, PCT cells were treated with triciribine for 24 h, washed and incubated in the absence of triciribine for 48 and 72 h, after which Akt phosphorylation on Thr308 and Ser473 was evaluated (Figure 6A). PCT cells did not display differences in Akt phosphorylation (Ser473 and Thr308) between control and recovery conditions (Figure 6A). Similarly, PCT cells exposed to myxothiazol 48 h after triciribine removal (Trici + 48 h + myxo) showed no resistance and died at the same level as the untreated cells, demonstrating the reversibility of the triciribine protective effect (Figure 6B).

## 3. Discussion

The success of kidney transplantation is seriously compromised by the nefarious effects of I/R, which is mandatory in the surgical procedure. Although a growing number of studies emphasize the pathological mechanisms of renal I/R injury, a satisfactory strategy to avoid undesirable effects of I/R has yet to be found. In this way, numerous studies have described the usefulness of many compounds when used as a pre- or per-conditioning I/R treatment. From a molecular point of view, it was clearly shown that several of these compounds required activation of PI3K/Akt and AMPK pathways to be effective. Nevertheless, none of these studies investigated the effect of PI3K/Akt inhibition alone on kidney sensitivity to I/R. In our work, we postulated that activation of Akt was not mandatory to withstand anoxia. Indeed, using GC7, a specific inhibitor of eIF5A hypusination and a well-characterized in vivo protective agent against renal I/R [41], we have shown that Akt is not activated but rather inhibited by GC7 treatment, with decreased phosphorylation at both serine473 and threonine308 residues. In addition, we have successfully used several Akt inhibitors as preconditioning agents and demonstrated their high effectiveness in protecting kidney cells from anoxia and mitotoxic drug exposure.

In search of underlying mechanisms related to Akt inhibition, we found a severe inhibition of lactate secretion without impairing mitochondrial activity in the preconditioning step. This is not surprising since Akt controls the glucose supply to the cells especially in response to insulin. This switch in energetic substrate could be beneficial for the cell in the context of ischemia. In fact, we can assume that cells treated with Akt inhibitors prefer to use an alternative energy substrate instead of glucose. Consequently, glucose can be redirected to the pentose phosphate pathway instead of glycolysis and thus participate in an increase in NADH production, which is a key cofactor in the antioxidant defense of the cell. Then, when cells were exposed to anoxia, regardless of the Akt inhibitor treatment, they showed a large increase in lactate secretion, demonstrating an increase in glycolysis, a classical cell response to a lack of oxygen. At this moment, we can suspect that cells contain a strong pool of NADH that can be used to protect cells from reactive oxygen species produced after ischemia and possibly later after reperfusion.

In contrast to glycolysis, oxidative phosphorylation was not influenced by triciribine pretreatment, except for a decrease in maximum mitochondrial oxygen consumption, which indicates a lower disposal in the respiratory chain when Akt is inhibited under normoxia. We did not measure these parameters under anoxia, but we can assume that oxygen deprivation led to a continuous decrease in mitochondrial activity, which is also the main source of mitochondrial ROS production. To mimic mitochondrial activity inhibition associated with ROS production, we treated cells with myxothiazol, an irreversible inhibitor of Complex III able to blunt mitochondrial activity. Interestingly, using this approach, we first demonstrated that triciribine-treated kidney cells became independent of mitochondria energy production and that this was accompanied by a decrease in mitochondrial ROS production due to the mitotoxic drug. Altogether, these results seem to demonstrate that preconditioning cells with Akt inhibitors first led to a decrease in glycolysis and then, when they were submitted to anoxia, allowed a shift of their metabolism from aerobic oxidative phosphorylation to anaerobic glycolysis without mitochondrial ROS production. This mechanism could alone be accountable for anoxia resistance due to Akt inhibition through preconditioning.

In line with this metabolic adaptation, Akt in cancer cells has been described as being influenced by ROS and can influence ROS production [46]. In this way, ROS activates Akt to inhibit HIF activity [47]. The HIF pathway is activated by oxygen deprivation and mediates metabolic adaptation and cell survival, playing also a central role in I/R response and adaptation [48,49,50]. The main metabolic role of the HIF pathway is to promote glucose oxidation and lactate production, which enables the cell to switch from aerobic to anaerobic metabolism. Thus, preventing HIF inhibition by ROS via inhibition of Akt at the time of ischemia can be highly beneficial for cells to adapt their metabolism to this stress [48]. Moreover, it has been shown that Akt/GSK3β pathway is involved in mitochondrial dysfunction leading to apoptosis of osteoblasts, and inhibition of this pathway prevented cell death [51]. This event was also mediated by ROS production [51].

Our results are not in accordance with the previous knowledge about the Akt pathway. Indeed, Akt is known to mediate cell survival in response to growth factors and cytokines and to prevent apoptosis induced by various types of stress, including I/R [30,52]. Among its functions, Akt is able to increase the activity of the mitochondrial-linked hexokinase by phosphorylation and, thus, to increase both glycolysis and the pyruvate used by oxidative phosphorylation [53]. This control of hexokinase is essential for the inhibition of apoptosis by Akt [54,55]. This role of Akt only makes sense if a cell’s energy metabolism is centered on glucose oxidation and oxidative phosphorylation. The kidney cells of the proximal tubule displayed an aerobic metabolism that preferred amino acids and lipids instead of glucose [45]. Indeed, as the main function of these cells is to reabsorb glucose from the urine into the plasma, they do not consume glucose for themselves and only barely used it through anaerobic glycolysis [13]. Thus, we can assume that this function of Akt linked to mitochondrial metabolism is not displayed by cells that are energetically independent of glucose, such as proximal convoluted tubule cells of the kidney.

Finally, Akt has been linked to apoptosis resistance in various cells and tissues, involving multiple and different pathways such as GSK3β [38,51], AMPK [56] or mTOR [57,58] and various targets such as HIF-1 [14], and hexokinases [55]. Unfortunately, we have not been able to determine the downstream pathway(s) that are modulated by the inhibition of Akt. Indeed, none of these pathways were involved in anoxia resistance displayed by PCT cells following GC7 or triciribine preconditioning (data not shown and [41,45]). As mentioned above, little was described about the role of Akt in these works regardless of drug treatment. In addition, many approaches have been described in the case of cardiomyocytes protection to I/R in which the role of the RISK signaling pathway has been clearly established [16], and even when Akt is expressed by all cells, it does not display the same functions and sensitivities to stimuli. Energetic metabolism is a critical factor in their function and cardiomyocytes, such as neurons, which display a glucose-dependent metabolism very different from proximal renal cells.

Another crucial characteristic of I/R management is the timing and the duration of treatments. Here, we have demonstrated a new and unexpected protection from anoxia after 24 h preconditioning by Akt inhibitors. We can assume that a later or shorter inhibition of Akt will lead to different results. Indeed, we have shown here that inhibition of Akt was only decreased during anoxia to protect cells from anoxia. As discussed previously, Akt inhibition appears clearly detrimental to cells submitted to a stress, including I/R [14,16,52]. In this context, we suspect that inhibiting Akt before the stress does not trigger a protective pathway but rather drives cells through an adaptation to I/R. This could be related to the concept of hormesis that characterizes ischemic preconditioning [23,26].

## 4. Materials and Methods

### 4.1. Reagents

Inhibitors, buffer solutions, fetal bovine serum (FBS) and other culture reagents were from Sigma-Aldrich Chimie (Merck, Saint-Quentin-Fallavier, France). N-guanyl-1,7-diaminoheptane (GC7) was synthesized by AtlanChim Pharma (Saint-Herblain, France) according to the method previously described [59].

### 4.2. Cell Culture

Renal proximal convoluted tubule cells (PCT) were obtained from primary cultures of murine proximal tubule segments, immortalized with pSV3neo vector and were cultured as previously described [60,61]. Cultures were classically maintained in a 5% CO2/95% air water-saturated atmosphere in M1 medium (DMEM/F12, Glutamine, SVF, EGF, T3, dexamethasone, ITS, G418). All experiments were performed the day after cell confluence. Minimum media (MM) is an isotonic solution devoid of nutrients: Tyrode′s solution in mM (NaCl 130, KCl 5, Hepes 10, MgCl_2_ 1, CaCl_2_ 1.8) as previously used [62]. For anoxia experiments, cells were cultivated at 37 °C in an airtight chamber subjected to a 100% N_2_ atmosphere. Oxygen deprivation was controlled using an OXYBABY^®^ apparatus to reach a concentration <0.1% (Wittgas, WITT, Morsang-sur-Orge, France).

### 4.3. Cell Metabolism Analysis

Oxygen consumption rate (OCR) and extracellular acidification rate (ECAR) of cells were measured with an XF24 Extracellular Flux Analyzer (Agilent Technologies, Seahorse Bioscience, Les Ulis, France). Uncoupled and maximal OCR were determined using oligomycin (1.2 µM) and FCCP (1 µM), respectively. Rotenone and Antimycin-A (2 µM each) were used to inhibit mitochondrial respiration. All parameters were calculated as described previously [63].

### 4.4. Cell Survival Analysis

Cell viability was assessed by staining cells with a mixture of Calcein-AM (1 µM, Invitrogen, Thermofisher Scientific, Illkirch-Graffenstaden, France) and Ethidium homodimer (4 µM, Sigma-Aldrich) for 45 min at 37 °C. Cells were then washed two times with PBS before imaging. Fluorescent micrographs were recorded using an observer D1 microscope (Zeiss, Iena, Germany) and analyzed using ImageJ software.

### 4.5. Protein Analysis

Whole proteins from cells were prepared using TNET lysis buffer (25 mM Tris-Cl (pH 7.4), 100 mM NaCl, 1 mM EDTA, 1% Triton X-100, 0.5% Nonidet P40, 1 × protease inhibitor cocktail and 1 × Phosphostop mix (Roche Diagnostics, Meylan, France)). Crude lysate was centrifuged at 10,000× *g* (30 min, 4 °C) to eliminate undisrupted cells as well as membranes and nuclei. Supernatant containing cytosolic proteins was preserved for analysis.

Protein concentration was evaluated by BCA assay (PIERCE, Thermofisher Scientific) and blotted using SDS-PAGE basic protocol and Mini-PROTEAN^®^ TGX™ Precast Protein Gels (BioRad, Marnes-la-Coquette, France). Primary antibody incubation was performed overnight at 4 °C (1:1000, bovine serum albumin 3%, p-Akt(ser473), p-Akt(thr308), pan Akt) and then with adequate HRP-conjugated secondary antibodies (Jackson ImmunoResearch Europe, Ely, CB7, United Kingdom) (30 min, bovine serum albumin 3%, 1:10,000, RT). Detection was performed using Immobilon Western Chemiluminescent HRP Substrate (Millipore, Merck, Saint-Quentin-Fallavier, France) and Fuji apparatus. Band intensities were evaluated using Fiji Software and displayed in a scatter plot.

### 4.6. Biochemical Parameter Analysis

Lactate concentrations were evaluated by ion chromatography analysis. Cell supernatants and ion standard solutions were previously deproteinized by addition of acetonitrile (dilution 1:1 volume). Samples were strongly mixed and centrifuged at 12,000× *g* (10 min at 4 °C). Lactate concentrations of the supernatant were determined using an ion chromatography Dionex ICS-5000 plus system (Thermofisher Scientific). The system included an autosampler, pumps, eluent generator and conductivity detectors. The system was equipped with 2 eluent generator cartridges (Dionex EGC500KOH; Dionex EGC500MSA) and an anion column (IonPac CS17, 2 mm). Lactate concentrations were determined using Chromeleon software (Thermofisher Scientific) by measuring surface area of the peaks and were compared to the corresponding ion standard profiles. Results were normalized by cell protein content evaluated using BCA assay.

### 4.7. Oxidative Stress Measurement

For measurement of mitochondrial superoxide anion levels, cells were loaded with 5 µM of MitoSOX Red probe (Molecular probes, Thermofisher Scientific) for 10 min at 37 °C, washed 3 times with pre-warmed HBSS and finally lysed in water. Fluorescence intensity was determined using a SP2000 Xenius plate reader (SAFAS, Monaco, Monaco) (510/580 nm) and then normalized to the cell protein content evaluated by a BCA assay on the same lysate.

Intracellular oxidative stress was measured by incubating cells for 45 min at 37 °C with 5 µM of CM-H2DCFDA (Molecular Probes), a probe mainly sensitive to hydroxyl radical and peroxynitrite. After incubation, cells were washed 3 times with HBSS and lysed in 200 μL of water. Fluorescence intensities were determined on the lysates with an SP2000 Xenius plate reader (SAFAS) (490/520 nm) and then normalized to the cell protein content evaluated by BCA assay on the same lysate.

GSH cell contents were evaluated as previously described [64].

### 4.8. Statistical Analysis

Data were analyzed using GraphPad Prism 6 software. Data were analyzed by t-test (2 groups) or ANOVA followed by Tukey’s post hoc test (more than 2 groups) to assess statistical differences between experimental groups. Differences were considered statistically significant with *p* < 0.05 *, *p* < 0.01 ** or *p* < 0.001 ***. Scatter plots were used to display the different sets of data for individual values of independent experiments along with mean ± SD.

## 5. Conclusions

The RISK pathway, and more particularly Akt, has been involved in cell adaptation. Positive or negative modulation of Akt is dependent on the type of cell, the stress and the kinetics of the injury. Given Akt’s unique and central position at the crossroad between different activators and effectors, its modulation can possibly trigger a timely, precise adaptative response. For example, in highly dependent on oxygen and nutrients cells, such as proximal kidney cells, the first line of defense against ischemia could be related to Akt inhibition as we show in this study. This process would not be incompatible with a late, long-term activation of Akt necessary to restore physiological function. Furthermore, it occurs that the well-established protective effect of GC7 against ischemia is linked to an inhibition of Akt and that direct Akt inhibition leads to the same protective effect, probably through preconditioning to prepare the cells to withstand any future I/R stress.

## Data Availability

All original data are available from the corresponding author upon request.
